# Prevalence and Severity of Social Anxiety Symptoms and Their Relationship With Body Dysmorphic Symptoms

**DOI:** 10.7759/cureus.53436

**Published:** 2024-02-02

**Authors:** Ahmad N Alhadi, Musab A Alageel, Fahad A Alsuhaibani, Hussain M Alkaff, Musaid S Albawardi, Abdullah A Alfaifi, Turky B Duraihem, Faisal A Alhayes

**Affiliations:** 1 Department of Psychiatry, College of Medicine, King Saud University, Riyadh, SAU; 2 Saudi Basic Industries Corporation (SABIC) Psychological Health Research and Applications Chair (SPHRAC), College of Medicine, King Saud University, Riyadh, SAU

**Keywords:** validation, saudi arabia, arabic, body dysmorphic disorder, social anxiety disorder

## Abstract

Background

Social anxiety disorder (SAD) is a subtype of anxiety characterized by avoidance, fear, and physical symptoms such as dry mouth, sweating, palpitations, and blushing. SAD is one of the most common mental disorders. Body dysmorphic disorder (BDD) is a mental disorder marked by a distressing or impairing preoccupation with imagined or minor flaws in one's physical appearance. Both disorders share similar symptoms. No satisfactory data have been provided about the prevalence of social anxiety symptoms in our region. In our study, we measured the prevalence and severity of SAD symptoms among adults in Riyadh City, as well as the sociodemographic factors associated with it. Additionally, the correlation between SAD and BDD was assessed.

Methods

Our study is quantitative, observational, and cross-sectional. It was conducted by administering a translated Arabic version of the Severity Measure for Social Anxiety Disorder scale and BDD scales in five locations in Riyadh, which include two general hospitals and three shopping malls. Data were analyzed using the SPSS version 22 (IBM Corp., Armonk, NY, USA). Descriptive statistical data are presented through mean values, standard deviations, and percentages.

Results

A total of 752 responses were received, of which 509 (68.32%) were from females with a mean age of 30.12 years. The majority of the sample had a low to middle family income, with 64% earning less than $2555 monthly. The sample possessed a good educational level; 63% had a bachelor's degree or higher. Our study also shows that 233 subjects (30.98%) had scores indicating a moderate to severe form of SAD. Among these participants, 86 (36.9%) had scores indicating a moderate to severe form of BDD. There was a significant positive correlation between SAD and BDD (r = 0.496).

Conclusion

The prevalence of SAD was 30.98%, which is higher compared to Western countries. Low income, education, and female gender have roles in the disease condition. Moreover, there was a linear relationship between SAD and BDD.

## Introduction

Social anxiety disorder (SAD) is defined according to the fifth edition of the Diagnostic and Statistical Manual of Mental Disorders - Text Revision (DSM-5-TR) as "marked fear or anxiety about one or more social situations in which the individual is exposed to possible scrutiny by others" [1]. Therefore, escape and avoidance behaviors are frequent. Patients with SAD also experience physiological symptoms during anxiety attacks, including restlessness, stomach aches, blushing, palpitations, muscle tension, and sweating [2]. According to the DSM-5-TR, these symptoms last for six months or more and cause significant distress or impairment.

Forty-three epidemiological studies have estimated the prevalence of SAD to be around 7-12% in Western countries [2]. Specific symptoms of social phobia and sociodemographic factors were assessed among more than 13,000 adults in the United States; the results show that the mean age of onset was 15.5 years and that it is higher in females than in their male counterparts. SAD is more common among individuals who are less educated, single, younger, and from lower socioeconomic classes [3]. A cross-national study conducted in 1996 revealed that out of every 100 persons, 2.6 are socially phobic in the US, while this number was 0.5 in Korea. However, rates among females are always higher [4]. A recent study, which gathered data from 28 community surveys with more than 140,000 participants, found that being a young, unmarried female with lower education and income are factors associated with SAD [2].

Body dysmorphic disorder (BDD) is described in the DSM-5-TR as "preoccupation with one or more perceived defects or flaws in physical appearance that are not observable or appear slight only to others, accompanied by repetitive behaviors (e.g., mirror checking, excessive grooming, skin picking, or reassurance seeking) or mental acts (e.g., comparing one’s appearance with that of other people) in response to the appearance concerns" [1]. This excessive concern with physical appearance is considered to be on the obsessive-compulsive spectrum. It is also associated with high levels of occupational and social disability, including absenteeism, lost productivity, unemployment, and marital dysfunction [5]. Moreover, excessive visits to dermatology and plastic surgery clinics are common. Recognizable BDD behavioral patterns include mirror checking, seeking reassurance, constant comparing, excessive grooming, and camouflaging (e.g., using hats and makeup) [6].

A study interviewing 2,048 people to assess the prevalence of BDD in the US revealed that 2.4% (by gender: 2.5% of women, 2.2% of men) met the DSM-IV criteria for BDD [7]. Another study conducted in Germany in 2006, using the DSM-IV criteria, revealed a 1.7% prevalence of BDD. It was associated with lower financial income, lower rates of living with a partner, and higher rates of unemployment [8].

SAD and BDD share similar characteristics. However, studies comparing BDD to SAD have not reached a consensus on whether a relationship exists between the two disorders [9]. Avoidance, a significant symptom of BDD, can greatly increase the risk of isolation and occupational impairment [10]. Avoidance is also a major criterion for SAD.

In a study examining the relationship between SAD and BDD, 6.7% of a sample of 165 patients with SAD were found to meet the criteria for BDD [11]. Conversely, a study published in 2012 claims that there is no direct link between SAD and BDD [12]. No satisfactory data are provided about the prevalence of social anxiety and the severity of symptoms in the Middle East region. Hence, in this study, we measure the prevalence of SAD symptoms among adults in Riyadh city and the sociodemographic factors associated with the prevalence and severity of the symptoms. Based on the literature, we hypothesize that the prevalence of SAD symptoms among adults is high in all forms (mild, moderate, severe) and related to sociodemographic factors such as gender, age, education, and socioeconomic class. Additionally, one focus of our research is to determine if a relationship between SAD and BDD exists in our community. Portions of this article were previously presented as a poster at the 26th European Congress of Psychiatry, on March 3-6, 2018.

## Materials and methods

Study design 

The study is quantitative, observational, and cross-sectional. We selected some hospitals and shopping malls in Riyadh as our study settings. We chose seven clinics (internal medicine, plastic surgery, ophthalmology, psychiatry, ENT, dermatology, and primary care) within two hospitals (King Khalid University Hospital and King Abdulaziz University Hospital). Data were collected from December 2016 to March 2017. We distributed paper questionnaires to the individuals attending these places. These participants come from different regions of Saudi Arabia and represent various sociodemographic factors. Additionally, these places are crowded, which made it easy to find people who meet our inclusion criteria. We targeted adults (18 years old and above) who speak, read, and write in Arabic. The exclusion criteria were children and individuals who cannot speak, read, and write in Arabic.

Sampling technique

In the hospitals, we collected data using a random, systematic sampling technique. The patient lists in the clinics were our sampling frame, and we have selected the patients randomly from it, starting from the first patient to the next one with a fixed periodic interval of five patients. We also distributed our questionnaires to the patients' companions (if present) to better reflect the general population. We preferred hospitals for their well-organized environment, the cooperation of patients and their companions, and the ease and efficiency of applying a systematic random sampling technique in such settings. In shopping malls, due to the challenge of creating a sampling frame, we opted for a convenience sampling method. We invited anyone entering the shopping malls who met the inclusion criteria to participate.

Questionnaires

Data were collected by distributing the questionnaires to participants without a fixed schedule. We translated, adapted, and validated our SAD scale instruments (APA DSM-5 Severity Measure for SAD Scale) [13] using clear and user-friendly guidelines for translation and cultural adaptation [14]. These guidelines provide recommendations for translating scales to ensure reliable and valid versions. First, the original scale was translated into Arabic by two independent translators whose native language is Arabic. Both are fluent and well-experienced in the cultures of the two languages and have a deep understanding of medical terminology. Second, we compared the two translated versions and then adopted one final version from both. Third, two other translators, native in English, back-translated the new Arabic version into English. These translators are fluent in both English and Arabic, have exceptional knowledge of both cultures and are experienced in translating medical studies. Fourth, we compared the two back-translations with each other and with the original instrument, making necessary changes by consensus of the research team. This process produced our Arabic SAD scale. We chose the Yale-Brown Obsessive-Compulsive Scale Modified for Body Dysmorphic Disorder (BDD-YBOCS) to assess the prevalence of BDD and to determine the relationship between social anxiety and BDD [15]. The BDD scale was translated into Arabic by Dr. M. Alshahwan and will be published soon (personal communication). The questionnaire was paper-based and included questions to gather sociodemographic data (age, gender, education level, occupation, marital status, and income) (Appendix 1).

Pilot study

We performed a pilot study to assess the logistics of data collection, the suitability/clarity of the data collection tools, and to estimate the timing for data collection. The questionnaire was piloted on 30 subjects, with answering the questionnaire taking an average of 6 minutes. Additionally, one question was added to the BDD scale: "Do you feel unsatisfied or uncomfortable about anything in your appearance (face/body)? (Yes/No)." The data from the pilot study were not included in the main study.

Sample size

The sample size calculated was 254 based on the prevalence of symptoms of SAD of 12%, which has been estimated by a previous study [16], considering 5% precision, a confidence interval of 95%, and an accuracy of an estimate of 4% using this formula:

$N= Z^{2} \times (P) \times (1-P)\div d^{2} = 1.96^{2} \times 0.12 \times (1-0.12) \div 0.04^{2}= 254$

P = Prevalence of symptoms of SAD which is 12%.

Z = 1.96 for a 95% confidence interval.

d = The accuracy of the estimate, in this study was 4%.

Considering a non-response rate of 45%. The minimal target sample size was 368.

Ethical consideration

The study was approved by the IRB Committee in the Department of Family and Community Medicine at the College of Medicine, King Saud University (IRB# 27/03/1438 (MA7)). Every participant received and signed an informed consent form, which explained the purpose of the study and their right to withdraw at any time without any obligation towards the study team. Additionally, participants' anonymity was assured by not collecting identifying data; all participants were anonymous. No incentives or rewards were given to the participants.

Data analysis

Data were analyzed using SPSS version 22 (IBM Corp., Armonk, NY, USA). Descriptive statistical data are presented as mean values, SDs, and percentages. According to the manual of the used scales, we classified the severity of social anxiety symptoms using the average score from this formula: (raw sum × 10) / Number of items that were actually answered) into None = 0, Mild = 1, Moderate = 2 severe =3, and Extreme = 4. The severity of BDD symptoms was classified according to the average score of answered items into not present = 0, extremely mild = 1-9, mild = 10-15, moderately severe = 16-25, and severe = 26-48.

## Results

The total number of responses included in this study was 752, with 577 participants (77.73%) from shopping malls and 175 (23.27%) from hospitals. Hospital participants were categorized into six groups: Psychiatry (65, 37.14%), Plastic and Dermatology (48, 27.4%), Internal Medicine (37, 21.14%), Primary Care and Family Medicine (10, 5.71%), Ophthalmology (9, 5.14%), and Ear Nose Throat (ENT) (6, 3.43%). The demographic data are shown in Table 1.

**Table 1:** Demographic data. The data has been represented as N, %, mean ± SD.

Item	N (%)		
Age: mean (SD)	30.12 ±10.24		
Gender			
Male		236	(31.68%)
Female		509	(68.32%)
Marital Status			
Single		365	(51.12%)
Divorced		25	(3.50%)
Married		319	(44.68%)
Widow		5	(0.70%)
Occupation			
Unemployed		116	(20.86%)
Employed		357	(64.21%)
Student		83	(14.93%)
Family Income			
<5000 income		285	(41.01%)
5000-1000 income		161	(23.17%)
10000-20000		172	(24.75%)
>20000		77	(11.08%)
Education level			
Illiterate		8	(1.11%)
Intermediate/secondary graduate		258	(35.93%)
Bachelor graduate		400	(55.71%)
Master/ PhD		52	(7.24%)

**Figure 1:**
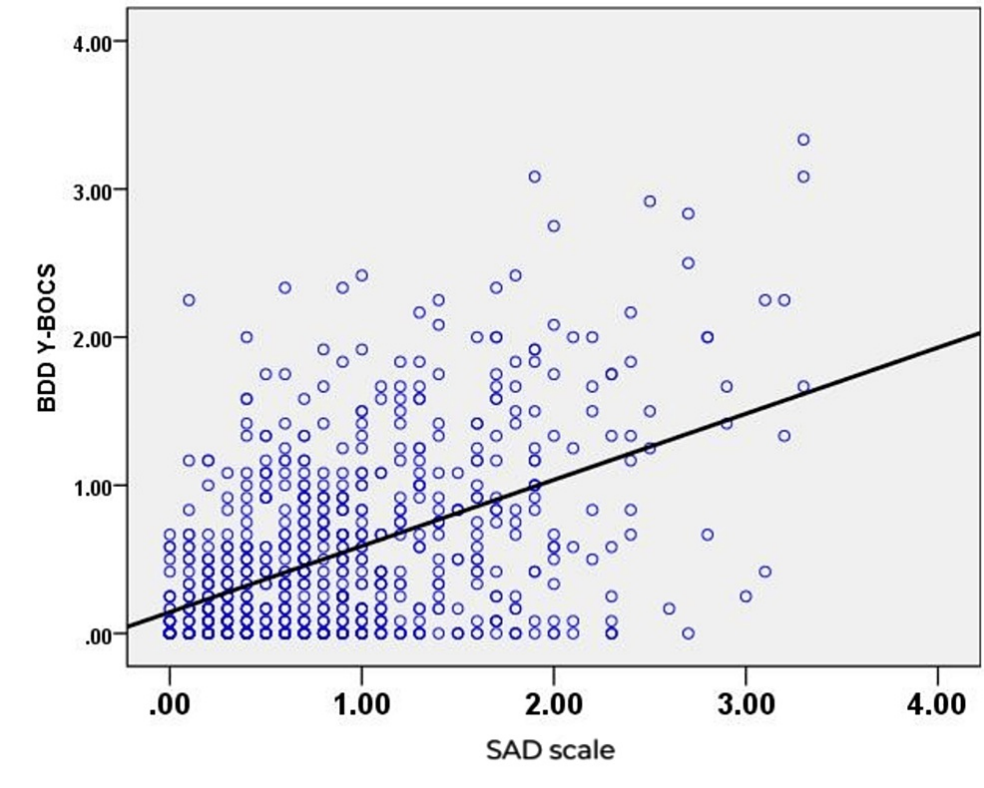
Correlation of social anxiety disorder symptoms (SAD) and body dysmorphic disorder (BDD). The data are represented as scores from the Social Anxiety Disorder Symptoms (SAD) scale and scores from the Body Dysmorphic Disorder (BDD) Y-BOCS scale. The Pearson Correlation Coefficient, r(750) = 0.496, p < 0.001, indicates a significant relationship. A p-value of less than 0.05 is considered significant.

The study also revealed that 233 subjects (30.98%) had social anxiety symptoms ranging from moderate to extreme, while 49 (6.52%) exhibited no symptoms. Of these, 470 (62.5%) had mild symptoms, 188 (25.5%) had moderate symptoms, 38 (5.05%) had severe symptoms, and seven (0.93%) had extreme symptoms. Additionally, 134 participants (17.82%) experienced BDD symptoms ranging from mild to severe, 200 (26.6%) had no symptoms, 418 (55.59%) had extremely mild symptoms, 114 (15.16%) had mild symptoms, 17 (2.26%) had moderately severe symptoms, and three (0.4%) had severe symptoms. The correlation between SAD and BDD symptoms was significant, r(750) = 0.496, p-value < 0.001 (Figure 1, Table 2). The relationships between SAD and BDD with age, gender, marital status, occupation, family income, and location are shown in Table 3.

**Table 2:** Correlation of social anxiety disorder symptoms (SAD) and body dysmorphic disorder (BDD). The data has been represented as number of cases (N) and percentages (%).

		BDD					Total N (%)
		None	Mild	Moderate	Severe	Extreme	
SAD	None	32	17	0	0	0	49 (6.5%)
	Mild	137	285	44	4	0	470 (62.5%)
	Moderate	26	103	51	7	1	188 (25%)
	Severe	5	12	17	4	0	38 (5%)
	Extreme	0	1	2	2	2	7 (1%)
Total N (%)		200 (26.6%)	418 (55.6%)	114 (15.2%)	17 (2.2%)	3 (0.4%)	752 (100%)

**Table 3:** The relationship of social anxiety and body dysmorphic disorder with demographic data. SAD: Social anxiety disorder; BDD: Body dysmorphic disorder; ENT: Ear, Nose and Throat. The data has been represented by Mean±SD. * p-value is considered significant at p<0.05.

		SAD		P-value	BDD		P-value
		Mean (SD)			Mean (SD)		
Age	Mean = 30.12 ±10.24	0.86	±0.68	<0.001*	0.53	±0.61	<0.001*
Gender	Male	0.74	±0.59	<0.001*	0.42	±0.50	<0.001*
	Female	0.92	±0.71		0.58	±0.65	
Marital status	Single	0.90	±0.70	0.062	0.57	±0.63	0.058
	Divorced	0.94	±0.77		0.57	±0.60	
	Married	0.80	±0.61		0.48	±0.57	
	Widow	1.36	±1.04		0.02	±0.04	
Occupation	Unemployed	0.89	±0.69	0.790	0.53	±0.60	0.603
	Employed	0.85	±0.67		0.51	±0.58	
	Student	0.83	±0.73		0.58	±0.63	
Family income	<5000 income	0.95	±0.69	0.032*	0.57	±0.60	0.223
	5000-1000 income	0.79	±0.62		0.56	±0.67	
	10000-20000	0.81	±0.69		0.47	±0.56	
	>20000	0.79	±0.72		0.45	±0.59	
Education level	Illiterate	0.56	±0.53	0.046*	0.53	±0.77	0.599
	Intermediate/secondary graduate	0.92	±0.71		0.55	±0.67	
	Bachelor graduate	0.85	±0.66		0.53	±0.58	
	Master/ hD	0.67	±0.57		0.42	±0.51	
Place	Malls	0.87	±0.68	0.011*	0.53	±0.60	0.122
	Internal Medicine OPD	0.69	±0.59		0.33	±0.43	
	Plastic and dermatology OPD	0.66	±0.55		0.59	±0.62	
	Psychiatry OPD	1.05	±0.75		0.58	±0.75	
	Primary care and Family medicine OPD	0.75	±0.42		0.38	±0.49	
	Ophthalmology	0.71	±0.38		0.52	±0.71	
	ENT OPD	1.43	±1.07		1.06	±0.60	

Females exhibited a higher prevalence of SAD symptoms compared to males (34% vs. 24%, respectively, p = 0.005). The severity categories for SAD among females and males are shown in Table 4.

**Table 4:** Symptoms severity of social anxiety disorder by gender.

	Male		Female	
	N	%	N	%
None	15	6.36	34	6.68
Mild	164	69.49	300	58.94
Moderate	48	20.34	139	27.31
Severe	8	3.39	30	5.89
Extreme	1	0.42	6	1.18
Total	236	100.00	509	100.00

Table 5 presents the test-retest reliability for the scales used, based on 50 participants who agreed to complete the questionnaire twice, one week apart. The reliability analyses for the APA SAD and BDD-YBOCS questionnaires yielded Cronbach’s alpha values of 0.859 and 0.914, respectively.

**Table 5:** Reliability analysis of the APA SAD questionnaire. APA SAD; American Psychiatric Association Social Anxiety Disorder severity measure scale. The data are represented as scale mean, scale variance, corrected item-total correlation, Cronbach's alpha, p-value, and Spearman's rho correlation coefficient. A p-value of less than 0.05 is considered significant.

Item number	Statement	Scale Means if Item Deleted	Scale Variance if Item Deleted	Corrected Item-Total Correlation	Cronbach's Alpha if Item Deleted	Test-retest reliability	
						Test-retest reliability p-value	Spearman's rho Correlation Coefficient*
Q1	Felt moments of sudden terror, fear, or fright in social situations	7.42	38.97	0.57	0.846	1.00	1.00
Q2	Felt anxious, worried, or nervous about social situations	7.02	36.99	0.59	0.844	1.00	1.00
Q3	Had thoughts of being rejected, humiliated, embarrassed, ridiculed, or offending others	7.55	37.76	0.58	0.845	1.00	1.00
Q4	Felt a racing heart, sweaty, trouble breathing, faint, or shaky in social situations	7.67	38.63	0.58	0.845	1.00	1.00
Q5	Felt tense muscles, felt on edge or restless, or had trouble relaxing in social situations	7.7	37.98	0.64	0.84	1.00	1.00
Q6	Avoided, or did not approach or enter, social situations	7.55	37.75	0.57	0.845	1.00	1.00
Q7	Left social situations early or participated only minimally (e.g., said little, avoided eye contact)	7.42	36.6	0.62	0.841	1.00	1.00
Q8	Spent a lot of time preparing what to say or how to act in social situations	7.25	37.43	0.53	0.849	1.00	1.00
Q9	Distracted myself to avoid thinking about social situations	7.41	36.92	0.59	0.844	1.00	1.00
Q10	Needed help to cope with social situations (e.g., alcohol or medications, superstitious objects)	7.93	41.16	0.4	0.858	1.00	1.00

## Discussion

The current study aimed to measure the prevalence and severity of social anxiety symptoms among adults in Riyadh as a primary objective. This study was motivated by the lack of epidemiological information about this common, underdiagnosed, and treatable condition in our society. Our results show the prevalence of extreme, severe, moderate, and mild forms of social anxiety symptoms to be 1%, 5%, 25%, and 62.5%, respectively. We found that the prevalence of social anxiety in Riyadh is 31% (moderate to extreme), which is higher than what we hypothesized. In a study conducted in a secondary school in Abha, Saudi Arabia, the prevalence of social anxiety symptoms was found to be 45% [17]. According to research that included over 9,000 participants in the US, the lifetime prevalence among adults is about 12.1% [18]. Moving regionally to Eastern countries, 9.3% of Egyptian university students were diagnosed with social anxiety based on a study that involved 300 undergraduate students [19]. Social anxiety in the Iranian population has been estimated at 10.1% [20]. The largest study on the prevalence of SAD, with 142,405 respondents in 28 countries, reported a lifetime prevalence of 4% [1]. Moreover, in a Brazilian study, the prevalence of social anxiety disorder was 28% [21]. An interesting finding, higher than previously reported, was found in a study that included seven countries with 6,825 young individuals, where the social anxiety prevalence was 36% [22]. The relatively high prevalence of social anxiety in our study can be accounted for in several ways. First, most of our participants were females (66%), and we found that they have a higher prevalence of social anxiety than males. If we had included equal numbers of males and females, we might have detected a lower prevalence. Second, our study was limited to Riyadh City, an urban area. Urban areas have been reported to have a higher prevalence of social anxiety than rural areas [20,23]. Third, this variation could be attributable to the use of different measuring tools. Fourth, Saudi society is collective rather than individualistic. Self-image and reputation are very important.

Concerning sociodemographic characteristics, our study demonstrates that age, gender, education level, and family income were statistically significant (P<0.05) factors in the prevalence and severity of SAD symptoms, consistent with the results of studies conducted outside of Saudi Arabia [3,21,24]. Moreover, our study found no significant association with marital status and employment, which does not match other studies [2,3]. Although most studies agree on the positive association between low family income and SAD, it is unclear whether low family income is a risk factor for SAD or if the relationship is the reverse [25].

We found that 134 (17.82 %) of our participants are likely suffering from BDD; which is relatively high compared to the literature. A study conducted in the eastern province of Saudi Arabia in patients visiting dermatology clinic found that the prevalence of BDD was 9.5% [26]. Another study in Makkah city found the prevalence of BDD to be 7.1% [27]. The Prevalence of BDD among female youth in Al-Madinah city was 14.68% [28]. A study in Lebanon found that the prevalence of BDD among adult females was 13.5% [29]. A meta-analysis examined twenty-two studies (n=7159) found the prevalence of BDD was 11.3% [30]. The relatively high proportion of participants likely suffering from BDD can be accounted for in several ways. First, our study included a large proportion of females (59.4%). Also, a considerable number of our data were collected in psychiatry, dermatology, and plastic clinics, where those with BDD are likely well-represented [11]. 

Some studies that assessed the correlation between SAD and BDD, and different methods have been applied to correlate BDD with SAD; some assessed the correlation based on the response to treatment, while others based their assessments on sociodemographic factors, onset, duration of the two diseases, and causative relationships. Researchers who have attempted to detect a link between these two disorders have reported contradictory findings. Some studies have reported a significant correlation; others propose that BDD is a subtype of SAD [9,12,23].

In our study, BDD and SAD symptoms were assessed using two questionnaires; then, we determined whether the increase in the severity of one disease is accompanied by a proportional increase in the other. To our knowledge, our study is the first in the Middle East to assess the correlation between SAD and BDD among the general population and on a good sample size (752 participants of Riyadh adults). In this population, a linear relationship between the severity of the two diseases has been identified. Among our participants, 233 (31%) showed moderate to severe symptoms of SAD; and among those, 86 have shown moderate to severe BDD. Some studies reported the same results. One study shows 11 participants (6.7%) of 165 patients with SAD to be also suffering from BDD [11]. Another study at Harvard University showed that people with BDD usually also suffer from different types of anxiety disorders, such as SAD [11]. A Chinese study found that there is a highly significant link between SAD and BDD among female medical students [31]. Also, there was a positive correlation between SAD and BDD based on a study that was done in Saudi Arabia among female medical students, although this did not reach statistical significance [32]. Some differences between our study and this study might explain the different results. For the BDD assessment, these authors used the Body Image Disturbance Questionnaire and BDD Symptomatology, while we used the BDD Modification of the Y-BOCS (BDD-YBOCS). For SAD assessment, these authors used the Social Interaction Anxiety Scale, while we used the APA DSM-5 Severity Measure for SAD Scale. Also, their study was done in a specific target population and in one place (female university students), while our study included both males and females and in multiple settings (hospitals and shopping malls) [32].

Our study had a larger proportion of female participants than male participants, and since the two disorders are seen more often in females, this could have affected the results of our study. Also, this study was done in Riyadh City, which restricts the generalization of the results to the whole population of Saudi Arabia. The used scales were severity measures of the disorders rather than diagnostic scales. Additionally, we could not apply a pure randomized method for our shopping mall participants because of the difficulties of making a sample frame. Moreover, we have not checked for the presence of real body distortion or malformation in the participants. Lastly, the scales were self-assessment tools instead of interview-based measures.

## Conclusions

We concluded that the prevalence of SAD among residents of Riyadh is high, surpassing figures reported in other countries and exceeding our initial expectations. Low income, female gender, and lower levels of education are associated with the disorder. More than a third of participants exhibiting a moderate to severe form of SAD also reported a moderate to severe form of BDD, indicating a linear correlation between SAD and BDD. Further prolonged prospective studies are required to clarify the relationship between BDD and SAD.

We recommend conducting the study in different regions of Saudi Arabia, which should be more comprehensive and representative and include more rigorous sample randomization.

## Appendices

Appendix 1

**Table 6:** Questionnaire.

Questionnaire						
Personal Information						
Gender	Male = 1	Female = 2				
Age	.............					
Marital Status	Single = 1	Divorced = 2	Married = 3	Widow = 4		
Monthly Family Income	1 = Less than 5000	2 = Between 5000 and 10,000	3 = Between 10,000 and 20,000	4 = More than 20,000		
Educational Level	Uneducated = 0	Primary/Middle School = 1	Secondary School = 2	Bachelor's Degree = 3	Master's Degree=4	Doctorate =5
Occupation	..............					
Severity Measure for Social Anxiety Disorder (Social Phobia) - Adult						
The following questions ask about thoughts, feelings, and behaviors that you may have had about social situations. Usual social situations include: public speaking, speaking in meetings, attending social events or parties, introducing yourself to others, having conversations, giving and receiving compliments, making requests of others, and eating and writing in public. Please respond to each item by marking (x) one box per row. During the PAST 7 DAYS, I have…						
Item		Never = 0	Occasionally = 1	Half of the time = 2	Most of the time = 3	All of the time = 4
1. felt moments of sudden terror, fear, or fright in social situations						
2. felt anxious, worried, or nervous about social situations						
3. had thoughts of being rejected, humiliated, embarrassed, ridiculed, or offending others						
4. felt a racing heart, sweaty, trouble breathing, faint, or shaky in social situations						
5. felt tense muscles, felt on edge or restless, or had trouble relaxing in social situations						
6. avoided, or did not approach or enter, social situations						
7. left social situations early or participated only minimally (e.g., said little, avoided eye contact)						
8. spent a lot of time preparing what to say or how to act in social situations						
9. distracted myself to avoid thinking about social situations						
10. needed help to cope with social situations (e.g., alcohol or medications, superstitious objects)						
Body Dysmorphic Disorder Modification of the Y-BOCS (BDD-YBOCS)						
Is there anything that bothers or makes you dissatisfied with your external appearance (face/body)?	Yes	No				
How much time do you spend thinking about this flaw in your appearance/body?	Nothing = 0	Less than an hour per day or occasionally (up to 8 times or less per day) = 1	From one to three hours per day or frequently (more than 8 times per day but most hours of the day are free from thoughts) = 2	From 3 to 8 hours per day or very frequently (more than 8 times per day and during most hours of the day) = 3	More than 8 hours per day or constantly occurring beyond the capacity to endure, rarely an hour passes without thinking = 4	
To what extent do thoughts about a flaw in your appearance/body affect your social and work life?	None = 0	Slight conflict with social or work activities, but general activities are not affected = 1	Clear conflict with social or work activities, but controllable = 2	Causes significant disruption in the performance of social or work activities = 3	Causes severe disruption = 4	
What is the level of disturbance resulting from your thoughts about a flaw in your appearance/body?	None = 0	Mild (sometimes), not annoying = 1	Moderate (usually), annoying but controllable = 2	Severe (most of the time) and very annoying = 3	Severe tension (constant) to the point of almost disabling = 4	
How much effort do you put into resisting these thoughts? In other words, how many times have you tried to reject or divert your mind from these thoughts?	These thoughts are few to the point that I don't need to resist them = 0	I try to resist these thoughts most of the time = 1	I exert little effort to resist these thoughts = 2	I am submissive to these thoughts with some hesitation = 3	I am completely submissive to these thoughts without hesitation = 4	
What is your level of control over thoughts related to a flaw in your appearance/body? What is your success rate in stopping or redirecting these thoughts?	Few thoughts and no need for control = 0	Good control (usually), I can stop or redirect these thoughts with some effort and focus = 1	Average control (sometimes), I can stop or redirect these thoughts with some effort and focus = 2	Simple control (rarely), I can stop or redirect these thoughts with some effort and focus = 3	I cannot control these thoughts = 4	
How much time do you spend on behaviors or activities related to your concern about your appearance/body?	Nothing = 0	Less than an hour per day or I engage in activities occasionally (no more than 8 times per day) = 1	From one to three hours per day or I engage in activities frequently (more than 8 times per day, but most hours are free from compulsive activities) = 2	More than 3 hours per day or I engage in activities very frequently (more than 8 times per day, and during most hours of the day) = 3	More than 8 hours per day or I engage in activities constantly (more than I can count, and rarely does an hour pass without engaging in activities) = 4	
How anxious are you if you refrain from engaging in behaviors or activities related to your appearance/body?	None = 0	Slight anxiety when refraining from actions = 1	Anxiety is apparent but bearable = 2	Clear and very disturbing anxiety = 3	Anxiety is apparent but bearable = 4	
How much effort do you exert to resist the urge to engage in behaviors or activities related to your appearance/body?	These behaviors are so few that I don't need to resist them = 0	I try to resist these behaviors most of the time = 1	I exert little effort to resist these behaviors = 2	Resigned to these behaviors with some hesitation = 3	Completely resigned to these behaviors without hesitation = 4	
What is the degree of control you have over your desire to engage in such behaviors or activities?	Complete control = 0	Usually, I control these behaviors = 1	Sometimes I can control these behaviors with difficulty = 2	I can delay these behaviors with difficulty, but they must be done eventually = 3	Rarely I can delay these behaviors, even for moments = 4	
To what extent are you convinced that there is a flaw in your appearance/body?	Convinced that there is no flaw in my appearance or body = 0	Acknowledge the absurdity of this idea but not convinced it's illogical = 1	Acknowledge with hesitation that this idea is illogical = 2	Convinced that this idea is logical = 3	No doubt that there is a flaw in my appearance or body = 4	
Have you avoided doing anything, going to a place, or being with anyone because of thoughts or behaviors related to a flaw in your appearance/body?	I don't avoid anything or anyone = 0	I avoid a little = 1	I avoid occasionally = 2	I avoid a lot = 3	I avoid everything and everyone = 4	
